# Combining radiotherapy with MEK1/2, STAT5 or STAT6 inhibition reduces survival of head and neck cancer lines

**DOI:** 10.1186/1476-4598-12-133

**Published:** 2013-11-05

**Authors:** Hanneke Stegeman, Johannes HAM Kaanders, Marieke MG Verheijen, Wenny JM Peeters, Deric L Wheeler, Mari Iida, Reidar Grénman, Albert J van der Kogel, Paul N Span, Johan Bussink

**Affiliations:** 1 Department of Radiation Oncology, Radboud University Nijmegen Medical Centre, PO Box 9101, 6500, HB Nijmegen, The Netherlands; 2 Department of Human Oncology, University of Wisconsin School of Medicine and Public Health, 1111 Highland Ave, Madison 53705, WI, USA; 3 Department of Otorhinolaryngology-Head and Neck Surgery and Department of Medical Biochemistry, Turku University Hospital and University of Turku, PO Box 52, FI-20521 Turku, Finland

**Keywords:** Radiation resistance, Head and neck cancer, Kinase inhibitors, STAT5, STAT6

## Abstract

**Background:**

Kinases downstream of growth factor receptors have been implicated in radioresistance and are, therefore, attractive targets to improve radiotherapy outcome in head and neck squamous cell carcinoma (HNSCC) patients.

**Methods:**

An antibody-based array was used to quantify the expression levels of multiple phospho-kinases involved in growth factor signaling in nine untreated or irradiated HNSCC lines. Radiosensitivity was assessed with clonogenic cell survival assays and correlated with the expression levels of the phospho-kinases. Inhibitors of the kinases that were associated with radiosensitivity were tested for their ability to increase radiosensitivity in the 3 most radioresistant HNSCC lines.

**Results:**

The basal expression of phosphorylated Yes, Src and STAT5A, and the expression after radiotherapy of phosphorylated AKT, MSK1/2, Src, Lyn, Fyn, Hck, and STAT6, were correlated with radiosensitivity in the panel of HNSCC lines. In combination with radiotherapy, inhibitors of AKT, p38 and Src Family Kinases (SFK) were variably able to reduce survival, whereas MEK1/2, STAT5 and STAT6 inhibition reduced survival in all cell lines. The combined effect of radiotherapy and the kinase inhibitors on cell survival was mostly additive, although also supra-additive effects were observed for AKT, MEK1/2, p38 and STAT5 inhibition.

**Conclusions:**

Kinases of the AKT, MAPK, STAT and SFK pathways correlated with radiosensitivity in a panel of HNSCC lines. Particularly inhibitors against MEK1/2, STAT5 and STAT6 were able to decrease survival in combination with radiotherapy. Hence, inhibitors against these kinases have the potential to improve radiotherapy outcome in HNSCC patients and further research is warranted to confirm this *in vivo*.

## Background

Radiotherapy is an integral part of the treatment of head and neck squamous cell carcinoma (HNSCC) and is successful in curing early-stage disease. However, the majority of HNSCC patients presents with locoregionally advanced disease for which cure rates remain relatively poor [1]. Increasing insight in the biological features of HNSCC tumors has resulted in the development of new therapeutic agents that target molecules important for survival after radiotherapy, including the Epidermal Growth Factor Receptor (EGFR). Combining these new agents with radiotherapy has already been successful in the clinic as a phase III study by Bonner et al. [2] has shown that cetuximab, a monoclonal antibody against EGFR, improves survival in patients treated with radiotherapy. However, despite this effect, a significant proportion of the patients is resistant to EGFR-inhibition and does not benefit from the addition of cetuximab. One of the proposed resistance mechanisms is activation of other growth factor receptors [3-6]. Different growth factor receptors, such as EGFR, other members of the ErbB family and MET, activate similar downstream pathways [7]. Due to this redundancy in signaling networks, cells overexpressing multiple growth factor receptors can sustain survival signaling when one of the receptors is blocked. Therefore, it will be important to determine the common downstream pathways that are responsible for cell survival after radiotherapy as they will be more attractive targets to overcome radioresistance than targeting one specific growth factor receptor.

Multiple kinase pathways downstream of growth factor receptors have already been implicated in radioresistance, including the RAS/RAF/ERK and the PI3-K/AKT pathways [8-10]. To identify kinases that can be targeted to increase radiosensitivity in HNSCC, it will be important to explore multiple pathways. In this study, we used an antibody-based array to quantify the expression levels of multiple phosphorylated kinases in a panel of HNSCC lines. The expression levels of these phospho-kinases were correlated with radiosensitivity. Expression levels were measured in untreated and irradiated cells as both basal activity and activity induced by radiation of a kinase could be important for cell survival after radiotherapy. Inhibitors of the kinases that were associated with radiosensitivity were tested for their ability to enhance the radiotherapy effect in HNSCC. We identified several kinase inhibitors that have the potential to increase radiosensitivity of tumors and thereby improve the outcome of HNSCC patients.

## Materials and methods

### Cell lines and chemicals

Nine human head and neck squamous cell carcinoma cell lines (UT-SCC lines, generated by R.G., University of Turku) were used in this study. The characteristics of the cell lines are shown in Table 1. Cell lines were not further authenticated or tested. Cells were cultured in T75 culture flasks, under humidified conditions (37°C, 5% CO_2_), and passaged weekly or twice weekly in DMEM containing 2 mM L-glutamine, 1% non-essential amino acids, 20 mM Hepes, 10 units/ml penicillin, 10 units/ml streptomycin, and 10% fetal bovine serum. The following kinase inhibitors and concentrations were used (concentrations were chosen on the basis of effectiveness described in the literature): Src Family Kinase inhibitor dasatinib (100 nM, LC Laboratories, Woburn, MA, USA) [11], AKT inhibitor MK-2206 (2 μM, Selleckchem, Houston, TX, USA) [12], MEK1/2 inhibitor U0126 (10 μM, Merck Millipore, Billerica, MA, USA) [8], p38 inhibitor SB203580 (10 μM, Selleckchem) [13], STAT5 inhibitor 573108 (100 μM, Merck Millipore) [14], and STAT6 inhibitor leflunomide (100 μM, Sigma, St Louis, MO, USA) [15].

**Table 1:** Characteristics of UT-SCC cell lines

**Cell line**	TNM*	**Primary tumor location**	**Type of lesion**	**Grade**	**SF4 ± SEM**
UT-SCC5	T_1_N_1_M_0_	Tongue	Primary	2	0.42 ± 0.03
UT-SCC8	T_2_N_0_M_0_	Supraglottic larynx	Primary	1	0.22 ± 0.02
UT-SCC15	T_1_N_0_M_0_	Tongue	Recurrence	1	0.31 ± 0.02
UT-SCC19A	T_4_N_0_M_0_	Glottic larynx	Primary	2	0.19 ± 0.02
UT-SCC24A	T_2_N_0_M_0_	Tongue	Primary	2	0.40 ± 0.02
UT-SCC29	T_2_N_0_M_0_	Glottic larynx	Primary	1	0.23 ± 0.01
UT-SCC38	T_2_N_0_M_0_	Glottic larynx	Primary	2	0.23 ± 0.03
UT-SCC40	T_3_N_0_M_0_	Tongue	Primary	1	0.33 ± 0.02
UT-SCC45	T_3_N_1_M_0_	Floor of mouth	Primary	3	0.28 ± 0.03

*TNM status of primary tumors according to the International Union against Cancer (1997).

Note: Grade: 1, well differentiated; 2, moderately differentiated; 3, poorly differentiated.

*SF4*, Surviving fraction after 4 Gy; *SEM*, Standard error of the mean.

### Human phospho-kinase antibody array

To determine levels of phospho-kinases at baseline and after radiotherapy, cells were harvested after no treatment or 1 h after a single dose of 4 Gy (320 KV, dose rate 3.1 Gy/min, X-RAD, RPS Services Limited, Surrey, UK). Cells were lysed using lysis buffer of the Human phospho-kinase array kit (ARY003, Proteome Profiler™, R&D Systems, Minneapolis, MN, USA) and protein was quantitated using a standard Bradford absorbance assay. The Human phospho-kinase array was performed according the protocol of the manufacturer. In this array, 46 capture antibodies are spotted in duplicate on nitrocellulose membranes. The capture antibodies were directed against the following antigens: AKT(S473), AKT(T308), AMPKα1(T174), AMPKα2(T172), Chk-2(T68), c-Jun(S63), CREB(S133), eNOS(S1177), ERK1/2(T202/Y204), T185/Y187), FAK(Y397), Fgr(Y412), Fyn(Y420), GSK-3α/β(S1/S9), Hck(Y411), HSP27(S78/S82), JNK pan (T183/Y185, T221/Y223), Lck(Y394), Lyn(Y397), MEK1/2(S218/S222, S222/226), MSK1/2(S376/S360), p27(T157), p27(T198), p38α(T180/Y182), p53(S15), p53(S46), p53(S392), p70 S6 kinase (T229), p70 S6 Kinase (T389), p70 S6 kinase (T421/S424), Paxillin(Y118), PLCγ-1(Y783), Pyk2(Y402), RSK1/2(S221), RSK1/2/3(S380), Src(Y419), STAT1(Y701), STAT2(Y689), STAT3(Y705), STAT4(Y693), STAT5a(Y699), STAT5a/b(Y699), STAT5b(Y699), STAT6(Y641), TOR(S2448), Yes(Y426) and β-catenin. In short, cell lysates were incubated with the membrane overnight. Thereafter, the membranes were incubated with a cocktail of biotinylated detection antibodies and streptavidin-HRP. Finally, proteins were detected using an ECL chemiluminescent system. To quantify expression levels, the integrated optical density (IOD) of each spot was measured using ImageJ software (NIH, Bethesda, MD, USA). IOD values were corrected for background signal and to compare different membranes levels were normalized to those of the positive controls on each membrane. Both the absolute expression levels after radiotherapy as well as the relative levels (expression after radiotherapy/expression in control) after radiotherapy were quantified.

### Radiosensitivity: Clonogenic cell survival assays

Cells were irradiated with graded doses (2, 4, or 8 Gy) at room temperature. After 1.5-3 weeks, depending on the growth speed of the cell line, cells were stained with 0.5% crystal violet and colonies with more than 50 cells were counted. Clonogenic survival curves were fitted using the linear quadratic model and the surviving fraction after 4 Gy (SF4) was calculated using the α and β values obtained from the curve.

### Kinase inhibition: Clonogenic cell survival assays & western blot analyses

For clonogenic cell survival assays, cells were incubated with the kinase inhibitor for 16 h and then irradiated with 4 Gy. Thereafter, cells were treated with the kinase inhibitor for 72 h (total 88 h of treatment) and subsequently cells were incubated in drug free medium. After 1.5-3 weeks, cells were stained with crystal violet and colonies were counted. Survival fraction after combined treatment with 4 Gy and the kinase inhibitor was calculated by correcting for plating efficiency of the untreated control or by correcting for plating efficiency of cells treated with the inhibitor alone.

For western blot analyses, cells were treated with the inhibitor for 16 h followed by irradiation with 4 Gy and harvested 4 h after radiotherapy or 20 h after kinase treatment. Cells were lysed in RIPA buffer and protein was quantitated using a standard Bradford absorbance assay. Proteins (25 μg per lane) were separated by SDS-PAGE and blotted onto PVDF membrane. Membranes were incubated with the appropriate primary antibodies followed by incubation with HRP-conjugated antibodies. Finally, proteins were detected using chemiluminescence. Antibodies against the following antigens were used: p-p38(T180/Y182), pMEK1/2(S217/221), pMSK1(S376), pSFK(Y416), pSTAT6(Y641), pSTAT5(Y694), pAKT(S473), pERK1/2(T202/Y204), and HRP-conjugated goat-anti-rabbit IgG were purchased from Cell Signaling Technology (Beverly, MA, USA), HRP-conjugated goat-anti-mouse IgG was purchased from Santa Cruz Biotechnology (Santa Cruz, CA, USA), and α-tubulin was obtained from Calbiochem (San Diego, CA, USA).

### Statistics

Correlations between expression levels of phospho-kinases (absolute levels in control, and both absolute levels and relative levels after radiotherapy) and SF4 values were assessed using the Spearman correlation test. To determine additive effects of combined treatment, differences between survival after 4 Gy and 4 Gy + inhibitor were tested for significance using the Mann–Whitney test. To determine supra-additive effects of combined treatment, differences between survival after 4 Gy and 4 Gy + inhibitor corrected for effect of inhibitor alone were tested for significance using the Mann–Whitney test. Tests were performed using Prism (GraphPad Software, Inc., LA Jolla, CA, USA) or SPSS (SPSS, Chicago, IL, USA). P-values ≤0.05 were considered significant.

## Results

### Expression of phospho-kinases correlated with radiosensitivity in a panel of HNSCC cell lines

**Figure 1:**
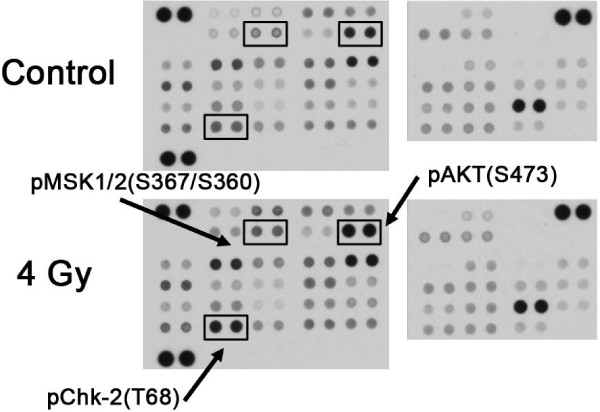
**Phospho-kinase array in control and irradiated UT-SCC24A cells.** Example of phospho-kinase array in untreated UT-SCC24A cells and 1 h after 4 Gy irradiation. Cell lysates were incubated with membranes containing capture antibodies (spotted in duplicate) against 46 kinase phosphorylation sites. Thereafter, the membranes were incubated with a cocktail of biotinylated detection antibodies and streptavidin-HRP. Proteins were detected using chemiluminescence and expression levels were quantified by measuring the integrated optical density (IOD) of each spot.

The radiosensitivity of 9 HNSCC cell lines was assessed with clonogenic survival assays after 0, 2, 4 and 8 Gy. Using the linear quadratic model, the surviving fraction after 4 Gy (SF4) was calculated for each cell line (Table 1). To determine which kinases are important for cell survival after radiotherapy in HNSCC, we quantified the expression of a panel of phospho-kinases using an antibody-based array in untreated and irradiated cells (Figure 1). The effect of radiotherapy on most phospho-kinases varied widely among cell lines, only the expression of p-Chk2 was increased in all cell lines after radiotherapy (Figure 1). The expression levels of multiple phospho-kinases were found to be significantly correlated with radiosensitivity (p-values ≤ 0.05 of spearman correlation test, Table 2). Only positive correlations were observed, indicating that higher levels of expression basally or after radiation for each of these proteins correlated with increasing radioresistance. For some phosphorylated kinases the basal expression level was correlated with radiosensitivity (Yes and STAT5A), whereas for others the expression level after radiotherapy (AKT, MSK1/2, Lyn, Fyn, Hck, STAT6). For phosphorylated Src both the basal expression level as well as the expression level after radiotherapy were correlated with radiosensitivity.

**Table 2:** Phospho-kinases correlated with radiosensitivity in HNSCC

**Phospho-kinase**	**Condition**	**Spearman correlation coefficient**	**P-value**
Src(Y419)	Expression in control	0.67	0.049
	Expression after RT	0.75	0.019
STAT5A(Y699)	Expression in control	0.70	0.036
Yes(Y426)	Expression in control	0.67	0.050
AKT(S437)	Relative expression after RT	0.67	0.050
MSK1/2(S376/S360)	Expression after RT	0.67	0.050
Lyn(Y397)	Expression after RT	0.70	0.036
Fyn(Y420)	Expression after RT	0.70	0.036
Hck(Y411)	Expression after RT	0.72	0.030
STAT6(Y641)	Expression after RT	0.67	0.050

RT: 4 Gy of radiotherapy.

Expression in control or after RT: absolute expression level in control or 1 h after RT.

Relative expression after RT: expression after radiotherapy divided by expression in control.

### Radiosensitizing effect of kinase inhibitors

**Figure 2:**
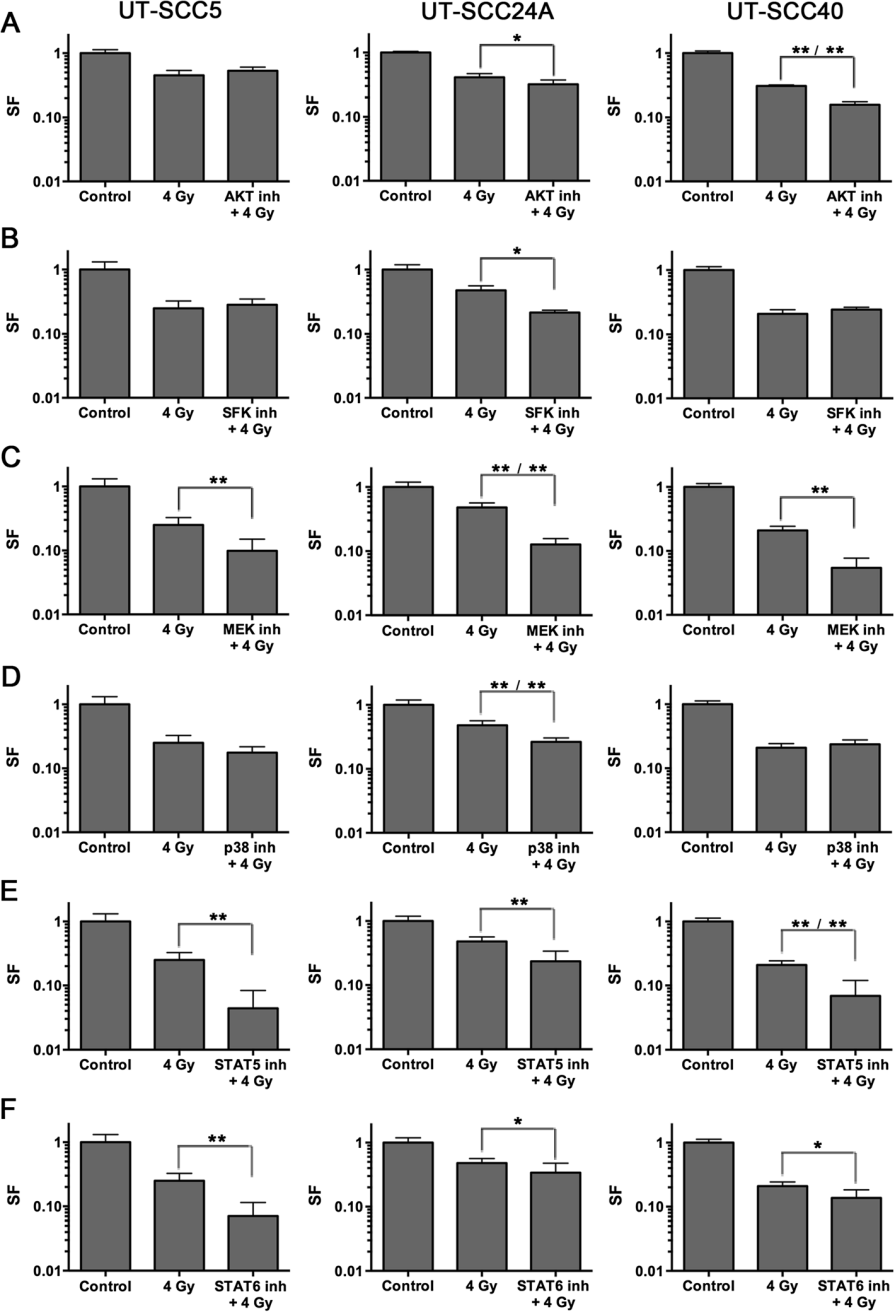
**Effects of kinase inhibitors on survival after radiotherapy.** UT-SCC5, 24A and 40 cell lines were treated with the inhibitor overnight (16 h), irradiated with 4 Gy and changed to drug free medium 72 h after irradiation. Survival was assessed via colony counting. **A)** AKT inhibitor: MK-2206 (2 μM), **B)** SFK inhibitor: dasatinib (100 nM), **C)** MEK1/2 inhibitor: U0126 (10 μM), **D)** p38 inhibitor: SB203580 (10 μM), **E)** STAT5 inhibitor: 573108 (100 μM), **F)** STAT6 inhibitor: leflunomide (100 μM). Survival fraction shown in graph was not corrected for the effect of the inhibitor alone. Differences between survival after 4 Gy and 4 Gy + inhibitor (additive effect, first asterisks) or 4 Gy and 4 Gy + inhibitor corrected for effect of inhibitor alone (supra-additive effect, second asterisks) were tested for significance using Mann–Whitney tests. *: p < 0.05, **: p < 0.01. Error bars represent SD.

**Figure 3:**
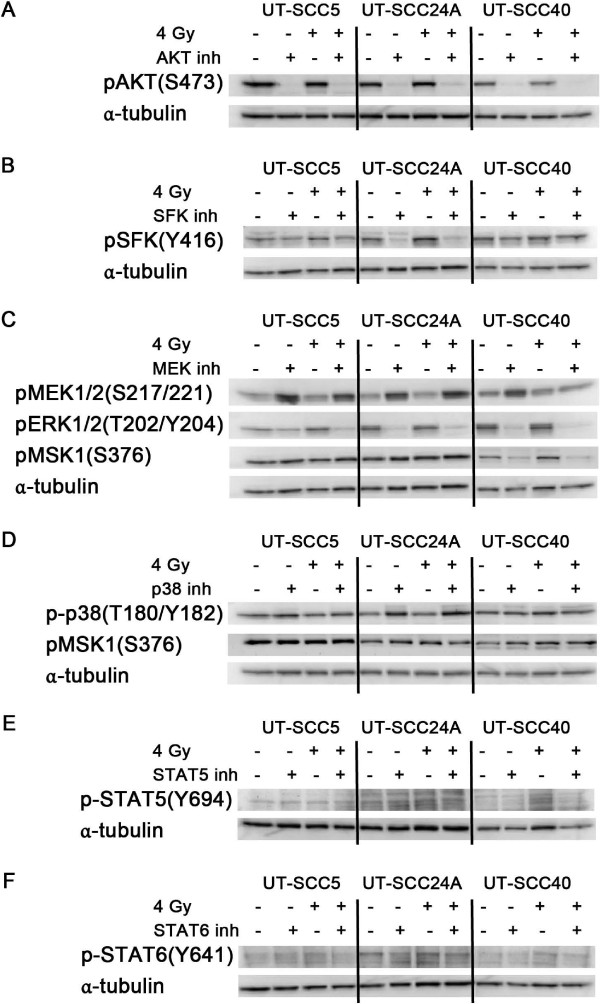
**Western blot analysis of phospho-kinases after treatment with inhibitor and/or radiotherapy.** UT-SCC5, 24A and 40 cells were incubated with the inhibitor overnight (16 h), irradiated with 4 Gy and harvested 4 h after radiotherapy treatment or 20 h after kinase treatment. **A)** AKT inhibitor: MK-2206 (2 μM), **B)** SFK inhibitor: dasatinib (100 nM), **C)** MEK1/2 inhibitor: U0126 (10 μM), **D)** p38 inhibitor: SB203580 (10 μM), **E)** STAT5 inhibitor: 573108 (100 μM), **F)** STAT6 inhibitor: leflunomide (100 μM). α-tubulin was used as loading control.

The significant correlation between the expression levels of these phosphorylated kinases and radiosensitivity indicates that the activity of these kinases might be important for cell survival after radiotherapy. Indeed, AKT and Src have been implicated in resistance to radiotherapy in HNSCC before [9,11] and were also found to be correlated with radiosensitivity in this study. Hence, these kinases might represent new targets to increase radiosensitivity in HNSCC. To test this hypothesis, clonogenic survival assays were performed with inhibitors against these various kinases in combination with radiotherapy in 3 UT-SCC lines with the highest SF4 values i.e. the most radioresistant tumor cell lines; UT-SCC5, 24A and 40 (Figure 2A-F). MK-2206, 573108 STAT5 inhibitor, and leflunomide were used to inhibit AKT, STAT5 and STAT6, respectively. Dasatinib was used to inhibit the kinases of the Src Family Kinase (SFK), which include Src, Yes, Lyn, Fyn and Hck. MSK1/2 can be activated via both the MEK/ERK pathway as well as the p38 pathway [16]. Therefore, both U0126 and SB203580 were used to inhibit MEK1/2 and p38, respectively, and thereby inhibit downstream MSK1/2. Next to the clonogenic survival assays, western blot analyses were performed on cells treated with the inhibitor and/or radiotherapy to determine the effects of the inhibitors on the phosphorylated kinases (Figure 3A-F).

As shown in Figure 2A, AKT inhibition significantly decreased survival after 4 Gy in UT-SCC24A (p = 0.030) and UT-SCC40 (p = 0.002). This effect was supra-additive in UT-SCC40 (p = 0.002). In all three cell lines AKT inhibition with or without radiotherapy clearly decreased pAKT levels (Figure 3A). SFK inhibition only decreased survival after 4 Gy in UT-SCC24A (p = 0.002), and this was not a synergistic effect (Figure 2B). Western blot analyses also showed only a clear decrease in pSFK levels in UT-SCC24A cells (Figure 3B). MEK inhibition significantly decreased survival after 4 Gy in all cell lines (p < 0.001), which was supra-additive in UT-SCC24A (p = 0.004) (Figure 2C). MEK inhibition increased pMEK1/2 levels in all cell lines (Figure 3C). In contrast, downstream pERK1/2 levels were decreased after MEK inhibition, indicating that the kinase activity of MEK1/2 was decreased despite a higher level of phosphorylated MEK1/2. However, this inhibition of ERK1/2 did only lead to reduced pMSK1 levels in UT-SCC40 (Figure 3C). Inhibition of p38 in combination with radiotherapy also led to a reduction of survival in UT-SCC24A (p = 0.002), which was a supra-additive effect (p = 0.002) (Figure 2D). Similar to what was seen using the MEK inhibitor, p38 inhibition did not lead to reduced p-p38 levels; rather p-p38 levels were increased in UT-SCC24A that showed a synergistic effect of p38 inhibition and radiotherapy (Figure 3D). However, no decrease in downstream pMSK1 levels were seen in any of the three cell lines after p38 inhibition indicating that the effect of p38 inhibition was not related to effects on MSK1 activity.

As shown in Figures 2E and 2F, both STAT5 and STAT6 inhibition led to a significantly decreased survival after 4 Gy in all cell lines (p < 0.01 and p < 0.05). For STAT6 inhibition this was only an additive effect, while STAT5 inhibition and 4 Gy had a supra-additive effect on cell survival in UT-SCC40. Both pSTAT5 and pSTAT6 levels were low and difficult to detect on western blot. Reduction of pSTAT5 was observed in UT-SCC40 and of pSTAT6 in UT-SCC5 and UT-SCC40 (Figure 3E,F).

## Discussion

In this study, an antibody-based array was used to determine which activated kinases involved in growth factor signaling were correlated with radiosensitivity in HNSCC. This screen resulted in multiple kinases of different pathways, which could be potential targets to increase radiosensitivity. Pathways known to be associated with radiosensitivity were found, including the RAS/RAF/ERK and the PI3-K/AKT pathways [8-10], validating our approach. In addition, kinases not known to be involved in radiosensitivity were identified, including STAT5 and STAT6. Moreover, inhibitors of these kinases were able to decrease survival after radiotherapy, particularly inhibitors against MEK1/2, STAT5 and STAT6. Hence, these kinases represent potential new targets to improve outcome after radiotherapy in HNSCC patients.

The PI3-K/AKT pathway has been shown to regulate important cell survival mechanisms that induce radioresistance, including DNA repair and proliferation [9]. Hence, inhibition of this pathway has been shown to be a major mechanism for the radiosensitizing effect of EGFR-inhibitors [17,18] and this is strengthened by the observation that activation of AKT has been implicated in resistance to EGFR-inhibition [19]. Here, we show that pAKT inhibition via MK-2206 can decrease survival after radiotherapy. This effect was supra-additive in one cell line, indicating that pAKT inhibition specifically decreased survival after radiotherapy in this cell line. However, pAKT inhibition did not decrease survival in all cell lines we tested, despite consistently good inhibition of pAKT levels (Figure 3A). Several mechanisms could explain this difference in radiosensitizing effect of MK-2206 between cell lines. Firstly, the importance of AKT activity for cell survival could differ between cell lines; for example also other kinases were highly expressed in resistant line UT-SCC5, and, therefore, inhibition of pAKT would not be deleterious for all cell lines. Moreover, numerous feedback systems are present between growth factor receptors and their downstream pathways, whereby inhibition of one kinase can lead to activation of receptors and consequently activation of other downstream pathways [20,21]. These feedback mechanisms can greatly impact the sensitivity of cells to kinase inhibitors. In addition, these mechanisms are likely differentially active between cell lines as they will be dependent on which receptors and kinases are (over)expressed or preferentially activated in a cell.

Several members of the family of Src kinases were also found to be correlated with radiosensitivity. SFKs have been shown to be involved in pathways that control cell division and survival [22,23] and Src has been implicated in AKT activation after radiotherapy [24]. However, dasatinib was only able to reduce survival after radiotherapy in UT-SCC24A cells in an additive way. This is in contrast with a recent study by Raju et al. [11], which showed that dasatinib enhances radiosensitivity in HNSCC cells via inhibition of radiation-induced DNA repair. A possible reason for this discrepancy is that due to differential sensitivity our panel of 3 cell lines was too small to detect the radiosensitizing effect of dasatinib. Namely, in the study of Raju et al. only 2 out of 6 cancer lines showed radiosensitization by dasatinib [11]. Nonetheless, these data together suggest that dasatinib can radiosensitize tumors, but that dasatinib is probably not effective in the majority of HNSCC patients.

In contrast to dasatinib, inhibition of MEK1/2 did result in decreased survival after radiotherapy in all cell lines, with a supra-additive effect in UT-SCC24A. MEK1/2 and its downstream kinases ERK1/2 have been implicated in radioresistance in HNSCC before, although the effect of pathway inhibition on radiosensitivity is inconsistent [8,25]. In this study, MEK1/2 inhibition was used to inhibit downstream phosphorylation of MSK1/2, which was correlated with radiosensitivity. Though clear inhibition of pERK1/2 was detected in all cell lines, pMSK1 was only decreased in UT-SCC40, which only showed an additive effect of MEK inhibition. Hence, these data suggest that the radiosensitizing effect of MEK inhibition is not regulated via MSK. Specific inhibition of MSK will be necessary to further investigate the role of MSK in radioresistance in HNSCC. Interestingly, the cell line that showed synergism between MEK inhibition and radiotherapy, also showed a synergistic effect of p38 inhibition. Also with this inhibitor no decrease of pMSK1 levels was observed. MEK and p38 both belong to the family of mitogen-activated protein kinases (MAPKs) [16]. Therefore, MEK and p38 may activate another common pathway that is important for survival after radiotherapy in UT-SCC24A cells, for example both MEK and p38 can activate MNK1 and thereby regulate mRNA translation [16].

Surprisingly, increased pMEK1/2 levels were observed in all cell lines after MEK inhibition (Figure 3C), and also p-p38 was increased by p38 inhibition in the cell line that showed decreased survival after radiotherapy (UT-SCC24A, Figure 3D). Upregulation of pMEK1/2 after MEK inhibition has also been observed by Turke et al. [21] and they attributed it to a negative feedback mechanism that activates an upstream signaling molecule. Indeed, we did observe decreased pERK1/2 levels indicating that MEK activity was decreased by the inhibitor despite increased pMEK1/2 levels. Accordingly, increased p-p38 levels after p38 inhibition in the sensitive cell line might indicate effective inhibition of p38 and its downstream pathways instead of increased activity of p38.

Members of the STAT family have been shown to be activated in epithelial tumors, including HNSCC, and are known to induce the transcription of genes involved in cell survival, proliferation and angiogenesis [26]. Activation of STAT5 has also been shown to contribute to tumor growth and resistance to cisplatin and EGFR-inhibition in HNSCC cell lines [27]. However, it has not been previously described that STAT5 and STAT6 correlate with radiosensitivity as we find in our study. Another member of the STAT family, STAT3, has been shown to be involved in resistance to radiotherapy [28]. Hence, our results indicate that also other STAT members play an important role in radiosensitivity in HNSCC. This is also indicated by a study of Lesterhuis et al. [29], who observed a trend toward a shorter progression-free survival for STAT6 expressing tumors in a cohort of HNSCC patients treated with radiotherapy only. More importantly, inhibition of STAT5 and STAT6 consistently decreased survival after radiation in all cell lines. Although these effects on survival were mostly additive, these data do suggest that inhibition of STAT5 and STAT6 has the potential to improve outcome after radiotherapy in a large proportion of HNSCC patients. However, our results have to be interpreted with caution. The effects of the inhibitors on pSTAT5 and pSTAT6 levels were small, although as we demonstrated for other kinases (MEK, p38), this does not necessarily reflect the activity of these kinases. Furthermore, leflunomide is not a very specific STAT6 inhibitor and we cannot exclude the possibility that the effect of leflunomide on cell survival is independent of STAT6 inhibition.

The specificity of the used inhibitors might be confirmed by performing knockdown experiments with siRNAs against the kinases identified in these experiments. However, also siRNAs are known to be prone to off-target effects and transfection of cells can induce stress responses that could have important consequences for the response to radiation of these cells. In addition, although specificity is an important issue, more important is that we show that multiple clinical available inhibitors have the potential to improve outcome after radiotherapy in HNSCC patients.

Altogether, mostly additive effects of the kinase inhibitors were observed in this study indicating that these inhibitors decreased tumor cell survival in general and not specifically after radiotherapy. Although a synergistic effect of a kinase inhibitor and radiotherapy would be preferred, combination therapies that result in reduced survival due to additive effects could still offer the promise of improving patient outcome after radiotherapy in the clinic. Especially when these additive effects occur in a large proportion of the patients. Recurrences after radiotherapy often occur from a few surviving clonogenic cells and this suggests that additional kill of clonogenic cells by a kinase inhibitor would contribute to local tumor control [30]. Further research will be necessary to assess the efficacy of these inhibitors to improve outcome after radiotherapy *in vivo* and ultimately in patients. Some of the concentrations used in our experiments to inhibit kinases were in the micromolar range and it can be questioned whether effective inhibitor concentrations will be obtainable *in vivo* and, hence, whether our findings can be directly extrapolated to the clinic. Our own group has already shown that combining dasatinib with radiotherapy results in a significant effect on growth delay in HNSCC xenografts, while either treatment alone has no effect on tumor growth [31]. In addition, clinical studies performed with dasatinib and MK-2206, have already shown to be able to effectively inhibit pSrc and pAKT, respectively [32,33]. Nonetheless, it will still need to be determined whether these inhibitors are also able to improve outcome after radiotherapy in the clinic. Lastly, the challenge for the future will be to determine which kinase pathway(s) are crucial for tumor cell survival in an individual patient and, hence, to determine which kinase inhibitor(s) will most likely be effective in that patient.

## Conclusion

Kinases of the PI3-K/AKT, MAPK, STAT and SFK pathways were shown to be correlated with radiosensitivity in HNSCC cells. Inhibitors of these kinases were able to decrease survival after radiotherapy, in particular MEK1/2, STAT5 and STAT6 inhibitors. Hence, kinase inhibitors have the potential to increase radiosensitivity of tumors and thereby improve the outcome of HNSCC patients after radiotherapy. However, as with inhibitors against growth factor receptors, tumor cell lines display differential sensitivity. Further research is warranted to increase insight in mechanisms involved in resistance to these kinase inhibitors and how they can be counteracted to increase the efficacy of these kinase inhibitors. Secondly, kinase inhibition should be tailored to the preferential signaling pathway activation of individual tumors.

## Competing interests

The authors declare that they have no competing interests.

## Authors’ contribution

HS designed and coordinated the project, performed the kinase arrays and drafted the manuscript. JHK, AJK, and JB obtained funding for this project and participated in its design and coordination, and drafted the manuscript. PNS helped with the statistical analyses and interpretation of the data and revised the manuscript. DLW and MI participated in the design and interpretation of the data. WJP and MMV designed and performed the cell culture experiments and performed the western blot analyses. RG provided the cell lines and revised the manuscript. All authors read and approved the final manuscript.

## References

[B1] Harari PM Ritter MA Petereit DG Mehta MP Chemoradiation for upper aerodigestive tract cancer: balancing evidence from clinical trials with individual patient recommendations Curr Probl Cancer 2004 28 7 40 10.1016/j.currproblcancer.2003.10.002 14688789

[B2] Bonner JA Harari PM Giralt J Azarnia N Shin DM Cohen RB Jones CU Sur R Raben D Jassem J Radiotherapy plus cetuximab for squamous-cell carcinoma of the head and neck N Engl J Med 2006 354 567 578 10.1056/NEJMoa053422 16467544

[B3] Wheeler DL Huang S Kruser TJ Nechrebecki MM Armstrong EA Benavente S Gondi V Hsu KT Harari PM Mechanisms of acquired resistance to cetuximab: role of HER (ErbB) family members Oncogene 2008 27 3944 3956 10.1038/onc.2008.19 18297114 PMC2903615

[B4] Schaefer G Haber L Crocker LM Shia S Shao L Dowbenko D Totpal K Wong A Lee CV Stawicki S A two-in-one antibody against HER3 and EGFR has superior inhibitory activity compared with monospecific antibodies Cancer Cell 2011 20 472 486 10.1016/j.ccr.2011.09.003 22014573

[B5] Xu H Stabile LP Gubish CT Gooding WE Grandis JR Siegfried JM Dual blockade of EGFR and c-Met abrogates redundant signaling and proliferation in head and neck carcinoma cells Clin Cancer Res 2011 17 4425 4438 10.1158/1078-0432.CCR-10-3339 21622718 PMC3138116

[B6] Stegeman H Kaanders JH van der Kogel AJ Iida M Wheeler DL Span PN Bussink J Predictive value of hypoxia, proliferation and tyrosine kinase receptors for EGFR-inhibition and radiotherapy sensitivity in head and neck cancer models Radiother Oncol 2013 106 383 389 10.1016/j.radonc.2013.02.001 23453541 PMC3627829

[B7] Guo A Villen J Kornhauser J Lee KA Stokes MP Rikova K Possemato A Nardone J Innocenti G Wetzel R Signaling networks assembled by oncogenic EGFR and c-Met Proc Natl Acad Sci USA 2008 105 692 697 10.1073/pnas.0707270105 18180459 PMC2206598

[B8] Affolter A Fruth K Brochhausen C Schmidtmann I Mann WJ Brieger J Activation of mitogen-activated protein kinase extracellular signal-related kinase in head and neck squamous cell carcinomas after irradiation as part of a rescue mechanism Head Neck 2011 33 1448 1457 10.1002/hed.21623 21928417

[B9] Bussink J van der Kogel AJ Kaanders JH Activation of the PI3-K/AKT pathway and implications for radioresistance mechanisms in head and neck cancer Lancet Oncol 2008 9 288 296 10.1016/S1470-2045(08)70073-1 18308254

[B10] Meyn RE Munshi A Haymach JV Milas L Ang KK Receptor signaling as a regulatory mechanism of DNA repair Radiother Oncol 2009 92 316 322 10.1016/j.radonc.2009.06.031 19615770 PMC2754282

[B11] Raju U Riesterer O Wang ZQ Molkentine DP Molkentine JM Johnson FM Glisson B Milas L Ang KK Dasatinib, a multi-kinase inhibitor increased radiation sensitivity by interfering with nuclear localization of epidermal growth factor receptor and by blocking DNA repair pathways Radiother Oncol 2012 105 241 249 10.1016/j.radonc.2012.08.010 23010482

[B12] Stegeman H Kaanders JH Wheeler DL van der Kogel AJ Verheijen MM Waaijer SJ Iida M Grenman R Span PN Bussink J Activation of AKT by hypoxia: a potential target for hypoxic tumors of the head and neck BMC Cancer 2012 12 463 10.1186/1471-2407-12-463 23046567 PMC3517352

[B13] Davies SP Reddy H Caivano M Cohen P Specificity and mechanism of action of some commonly used protein kinase inhibitors Biochem J 2000 351 95 105 10.1042/0264-6021:3510095 10998351 PMC1221339

[B14] Marwarha G Prasanthi JR Schommer J Dasari B Ghribi O Molecular interplay between leptin, insulin-like growth factor-1, and beta-amyloid in organotypic slices from rabbit hippocampus Mol Neurodegener 2011 6 41 10.1186/1750-1326-6-41 21651786 PMC3121598

[B15] Moynihan B Tolloczko B Michoud MC Tamaoka M Ferraro P Martin JG MAP kinases mediate interleukin-13 effects on calcium signaling in human airway smooth muscle cells Am J Physiol Lung Cell Mol Physiol 2008 295 L171 L177 10.1152/ajplung.00457.2007 18441092 PMC2494781

[B16] Roux PP Blenis J ERK and p38 MAPK-activated protein kinases: a family of protein kinases with diverse biological functions Microbiol Mol Biol Rev 2004 68 320 344 10.1128/MMBR.68.2.320-344.2004 15187187 PMC419926

[B17] Toulany M Kasten-Pisula U Brammer I Wang S Chen J Dittmann K Baumann M Dikomey E Rodemann HP Blockage of epidermal growth factor receptor-phosphatidylinositol 3-kinase-AKT signaling increases radiosensitivity of K-RAS mutated human tumor cells in vitro by affecting DNA repair Clin Cancer Res 2006 12 4119 4126 10.1158/1078-0432.CCR-05-2454 16818713

[B18] Kang KB Zhu C Wong YL Gao Q Ty A Wong MC Gefitinib radiosensitizes stem-like glioma cells: inhibition of epidermal growth factor receptor-Akt-DNA-PK signaling, accompanied by inhibition of DNA double-strand break repair Int J Radiat Oncol Biol Phys 2012 83 e43 e52 10.1016/j.ijrobp.2011.11.037 22516386

[B19] Wheeler DL Dunn EF Harari PM Understanding resistance to EGFR inhibitors-impact on future treatment strategies Nat Rev Clin Oncol 2010 7 493 507 10.1038/nrclinonc.2010.97 20551942 PMC2929287

[B20] Chandarlapaty S Sawai A Scaltriti M Rodrik-Outmezguine V Grbovic-Huezo O Serra V Majumder PK Baselga J Rosen N AKT inhibition relieves feedback suppression of receptor tyrosine kinase expression and activity Cancer Cell 2011 19 58 71 10.1016/j.ccr.2010.10.031 21215704 PMC3025058

[B21] Turke AB Song Y Costa C Cook R Arteaga CL Asara JM Engelman JA MEK inhibition leads to PI3K/AKT activation by relieving a negative feedback on ERBB receptors Cancer Res 2012 72 3228 3237 10.1158/0008-5472.CAN-11-3747 22552284 PMC3515079

[B22] Summy JM Gallick GE Src family kinases in tumor progression and metastasis Cancer Metastasis Rev 2003 22 337 358 10.1023/A:1023772912750 12884910

[B23] Yeatman TJ A renaissance for SRC Nat Rev Cancer 2004 4 470 480 10.1038/nrc1366 15170449

[B24] Kim MJ Byun JY Yun CH Park IC Lee KH Lee SJ c-Src-p38 mitogen-activated protein kinase signaling is required for Akt activation in response to ionizing radiation Mol Cancer Res 2008 6 1872 1880 10.1158/1541-7786.MCR-08-0084 19074832

[B25] Bonner JA Vroman BT Christianson TJ Karnitz LM Ionizing radiation-induced MEK and Erk activation does not enhance survival of irradiated human squamous carcinoma cells Int J Radiat Oncol Biol Phys 1998 42 921 925 10.1016/S0360-3016(98)00325-3 9845123

[B26] Lai SY Johnson FM Defining the role of the JAK-STAT pathway in head and neck and thoracic malignancies: implications for future therapeutic approaches Drug Resist Updat 2010 13 67 78 10.1016/j.drup.2010.04.001 20471303

[B27] Koppikar P Lui VW Man D Xi S Chai RL Nelson E Tobey AB Grandis JR Constitutive activation of signal transducer and activator of transcription 5 contributes to tumor growth, epithelial-mesenchymal transition, and resistance to epidermal growth factor receptor targeting Clin Cancer Res 2008 14 7682 7690 10.1158/1078-0432.CCR-08-1328 19047094 PMC3422894

[B28] Bonner JA Trummell HQ Willey CD Plants BA Raisch KP Inhibition of STAT-3 results in radiosensitization of human squamous cell carcinoma Radiother Oncol 2009 92 339 344 10.1016/j.radonc.2009.06.022 19616333 PMC5906031

[B29] Lesterhuis WJ Punt CJ Hato SV Eleveld-Trancikova D Jansen BJ Nierkens S Schreibelt G de Boer A van Herpen CM Kaanders JH Platinum-based drugs disrupt STAT6-mediated suppression of immune responses against cancer in humans and mice J Clin Invest 2011 121 3100 3108 10.1172/JCI43656 21765211 PMC3148725

[B30] Baumann M Krause M Dikomey E Dittmann K Dorr W Kasten-Pisula U Rodemann HP EGFR-targeted anti-cancer drugs in radiotherapy: preclinical evaluation of mechanisms Radiother Oncol 2007 83 238 248 10.1016/j.radonc.2007.04.006 17502118

[B31] Stegeman H Span PN Rijken PF Cockx SC Wheeler DL Iida M van der Kogel AJ Kaanders JH Bussink J Dasatinib Inhibits DNA Repair after Radiotherapy Specifically in pSFK-Expressing Tumor Areas in Head and Neck Xenograft Tumors Transl Oncol 2013 6 413 419 23908684 10.1593/tlo.13259 PMC3730016

[B32] Brooks HD Glisson BS Bekele BN Johnson FM Ginsberg LE El-Naggar A Culotta KS Takebe N Wright J Tran HT Papadimitrakopoulou VA Phase 2 study of dasatinib in the treatment of head and neck squamous cell carcinoma Cancer 2011 117 2112 2119 10.1002/cncr.25769 21523723 PMC3117018

[B33] Yap TA Yan L Patnaik A Fearen I Olmos D Papadopoulos K Baird RD Delgado L Taylor A Lupinacci L First-in-man clinical trial of the oral pan-AKT inhibitor MK-2206 in patients with advanced solid tumors J Clin Oncol 2011 29 4688 4695 10.1200/JCO.2011.35.5263 22025163

